# Adapting to an increasingly stressful environment: Experimental evidence for ‘micro‐evolutionary priming’

**DOI:** 10.1111/1365-2656.70012

**Published:** 2025-02-19

**Authors:** Shuwen Han, Paul J. Van den Brink, Steven A. J. Declerck

**Affiliations:** ^1^ Department of Aquatic Ecology Netherlands Institute of Ecology (NIOO‐KNAW) Wageningen The Netherlands; ^2^ Department of Aquatic Ecology and Water Quality Management Wageningen University Wageningen The Netherlands; ^3^ Department of Biology, Laboratory of Aquatic Ecology, Evolution and Conservation KULeuven Leuven Belgium

**Keywords:** copper, eco‐evolutionary dynamics, experimental evolution, genetic adaptation, micro‐evolutionary adaptation, pollution, rotifer, stress

## Abstract

1. In many natural systems, animal populations are exposed to increasing levels of stress. Stress levels tend to fluctuate, and long‐term increases in average stress levels are often accompanied by greater amplitudes of such fluctuations. Micro‐evolutionary adaptation may allow populations to cope with gradually increasing stress levels but may not prevent their extirpation during acute stress events unless adaptation to low stress levels also increases their tolerance to acute stress.
2. We tested this idea, here called ‘micro‐evolutionary priming’, by exposing populations of the monogonont rotifer species *Brachionus calyciflorus* to four levels of copper stress (control, low, intermediate and high) during a multigenerational selection experiment. Subsequently, in a common garden experiment, we exposed randomly selected subsets of genotypes (clones) of each of these populations to low, intermediate and high copper levels and assessed their population growth performance across multiple generations.
3. Compared to populations with an exposure history to copper, genotypes of control populations suffered strong growth reductions when exposed to intermediate and high levels of copper, mainly as a result of high mortality rates. Remarkably, when exposed to low copper levels, fitness differences between genotypes of control populations and populations adapted to these low levels were very small, whereas the latter strongly outperformed the former at intermediate and high copper levels.
4. These results highlight the potentially strong but hitherto largely ignored impact of micro‐evolutionary priming on the performance of populations in a changing environment. We discuss the potential consequences of micro‐evolutionary priming for the persistence of populations and the spatial eco‐evolutionary dynamics of metapopulations.

## Graphical abstract

Our study shows that exposure to low stress makes zooplankton populations more tolerant to extreme stress. This phenomenon, here called micro‐evolutionary priming, is rarely studied in animals. We provide strong evidence for its existence and explore its implications for the fate of populations in an increasingly stressful world.

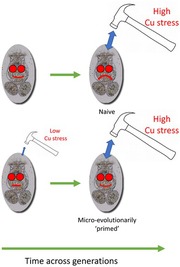

## 1 INTRODUCTION

**FIGURE 1:**
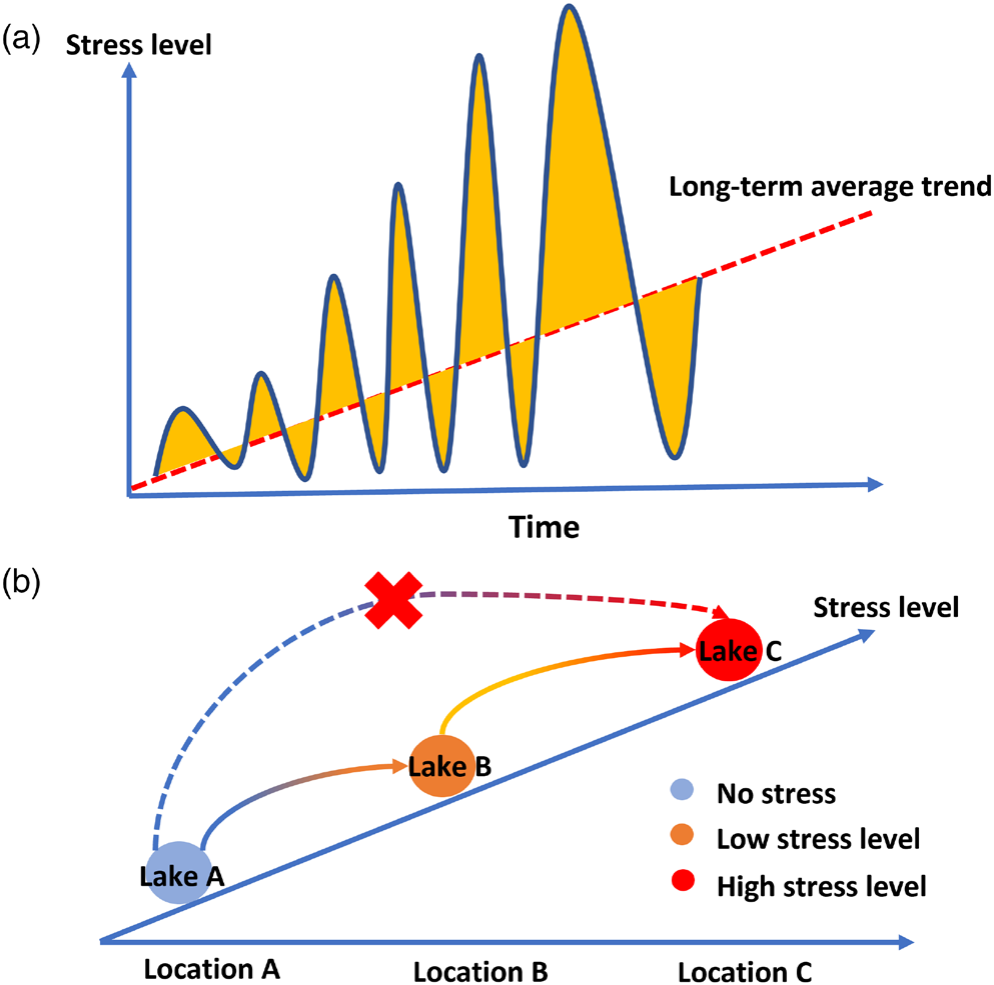
Hypothetical scenarios illustrating how micro‐evolutionary priming may affect the temporal and spatial dynamics of populations. (a) Increases in long‐term average stress are often accompanied by an increased amplitude of stress fluctuations. Through micro‐evolutionary priming, adaptation to low levels of stress may increase populations' persistence during episodes of acute stress. (b) Micro‐evolutionary priming may contribute to the spatial expansion of metapopulations. By allowing naïve populations to adapt to low‐stress levels, low‐stress sites may serve as stepping stones between stress‐free and high‐stress sites.

In many ecosystems, organisms are increasingly exposed to a broad range of stressors as a consequence of anthropogenic influences. Populations may be able to sustain growth under such conditions thanks to phenotypic plasticity and rapid micro‐evolutionary adaptation. Long‐term trends of gradually augmenting stress levels are often accompanied by an increased frequency and intensity of acute stress events (Figure 1a; Bernhardt et al., 2020; Easterling et al., 2000; Li, 2020). A relevant question is then to what extent a population's initial exposure to a low level of stress provides an increased ability to cope with much higher levels of stress.

Evidence for such ability is provided by different research fields. In ecotoxicology, exposure of organisms to low levels of toxicants is known to improve their ability to deal with considerably higher concentrations later in life (Agathokleous & Calabrese, 2020; Costantini et al., 2010; Sebastiano et al., 2022) or even in the next generations (transgenerational hormesis; Agathokleous et al., 2022; Brevik et al., 2018). Most evidence for the inheritance of such coping abilities seems to involve some form of epigenetic inheritance and pertains to only one or a few generations, although examples exist that span many more generations (Shi et al., 2021; Yue et al., 2021). Evidence of a different kind is provided by experimental evolution studies primarily with microbial organisms showing how multigenerational exposure to low stress levels can drive mutation‐based genetic adaptations leading to tolerance to higher stress levels. Well‐known examples of this phenomenon, which we here term ‘micro‐evolutionary priming’, are studies showing how bacterial adaptation to low antibiotic levels leads to resistance against much higher concentrations (Gullberg et al., 2011; Lagator et al., 2021; Sandegren, 2014; Wistrand‐Yuen et al., 2018). Similarly, experiments with yeast have demonstrated increased tolerance and persistence of populations under high‐stress levels after adaptation to low stressor levels (Gonzalez & Bell, 2013; Samani & Bell, 2010).

Evidence for micro‐evolutionary priming in animal populations is much scarcer. Although significant research has focused on rapid micro‐evolutionary adaptation in fluctuating environments (Bergland et al., 2014; de Villemereuil et al., 2020; King & Hadfield, 2019; Pfenninger & Foucault, 2022), few studies specifically address how adaptation to very low‐stress levels may enhance tolerance of animal populations to future acute stress events. Even in the absence of mutation micro‐evolutionary priming may nevertheless be expected if directional selection by low‐stress levels increases the prevalence of alleles that contribute to coping with much higher levels of stress (Gonzalez & Bell, 2013).

There is indeed circumstantial evidence supporting the concept of micro‐evolutionary priming in animals. In evolutionary ecotoxicology, genetic adaptation of populations to stress has been found to be associated with tolerance to stress levels higher than what they have adapted to, for example, in the case of metals (Khan et al., 2011; Vigneron et al., 2015), salts (Coldsnow et al., 2017) and pesticides (Weston et al., 2013). Similarly, using the critical thermal maximum (CT_max_) concept, studies of thermal adaptation have demonstrated an increased ability of populations to cope with extreme heat stress after a history of selection by moderately increased ambient temperatures (Brans et al., 2017; Diamond & Chick, 2018; Doorslaer et al., 2009; Geerts et al., 2015). Although these studies offer indications, they were not specifically designed to test for micro‐evolutionary priming. Instead, their primary objective was to demonstrate genetic adaptation to stressors, often focusing on populations adapted to stress levels that cannot be considered truly low or on populations with uncertain histories of exposure to acute stress events. Furthermore, they typically evaluate only one fitness component, such as mortality under near‐lethal stress. The latter limits their ability to assess the impact of micro‐evolutionary priming on multi‐generational population performance.

Although the ecological implications of local adaptation are increasingly recognized (Derry et al., 2019; Meek et al., 2023), it is quite remarkable that the idea of micro‐evolutionary priming has been tested and discussed to such a limited extent. Recognizing micro‐evolutionary priming in animals and its ecological implications could be crucial for enhancing the prediction, management and conservation of their populations in a rapidly changing world. In this study, we aim to test for micro‐evolutionary priming using an experimental evolution approach with a planktonic metazoan. To achieve this, we conducted a laboratory selection experiment in which replicate multiclonal rotifer populations were allowed to adapt to varying copper levels, ranging from low to high. This was followed by a common garden experiment in which we compared the population growth rates of genotypes of these populations to each of the copper levels applied in the selection experiment. Our study differs from other studies in that our design includes the adaptation of populations to low‐stress levels, that is, stress levels for which we observe only a very limited negative impact on population growth rates. A second important difference is that we do not evaluate the impact of this adaptation through acute toxicity tests, but rather on realized population growth across multiple generations. We also discuss some so far unappreciated consequences that micro‐evolutionary priming may have for the persistence and spatial dynamics of (meta)populations in a variable environment.

## 2 MATERIALS AND METHODS

For our experiment, we used genotypes (clones) from a natural population of the rotifer *Brachionus calyciflorus s.s*. (Pallas, 1766), one of four species of the *B. calyciflorus* species complex (Michaloudi et al., 2018; Zhang & Declerck, 2022). Monogonont rotifers are characterized by short generation times, a small body size, and a cyclical parthenogenetic reproduction mode alternating clonal with sexual propagation, features that make these organisms very well suited for laboratory evolution experiments and the study of genotype‐specific responses to environmental stressors (Declerck & Papakostas, 2017; Serra et al., 2019). Clonal lines were obtained during the year 2020 by hatching dormant propagules collected from the sediments of a freshwater pond (location: 52.02630°, 4.18355°, The Netherlands) and by establishing clonally reproducing populations in the laboratory. As dormant propagules are sexually produced, each clone can be considered genetically distinct. We applied microsatellite analysis using the primers developed by (Declerck et al., 2015) to assure that all genotypes used in our study belonged to the species *B. calyciflorus s.s*. Ethical approval for this study was not required.

### 2.1 Selection experiment

**FIGURE 2:**
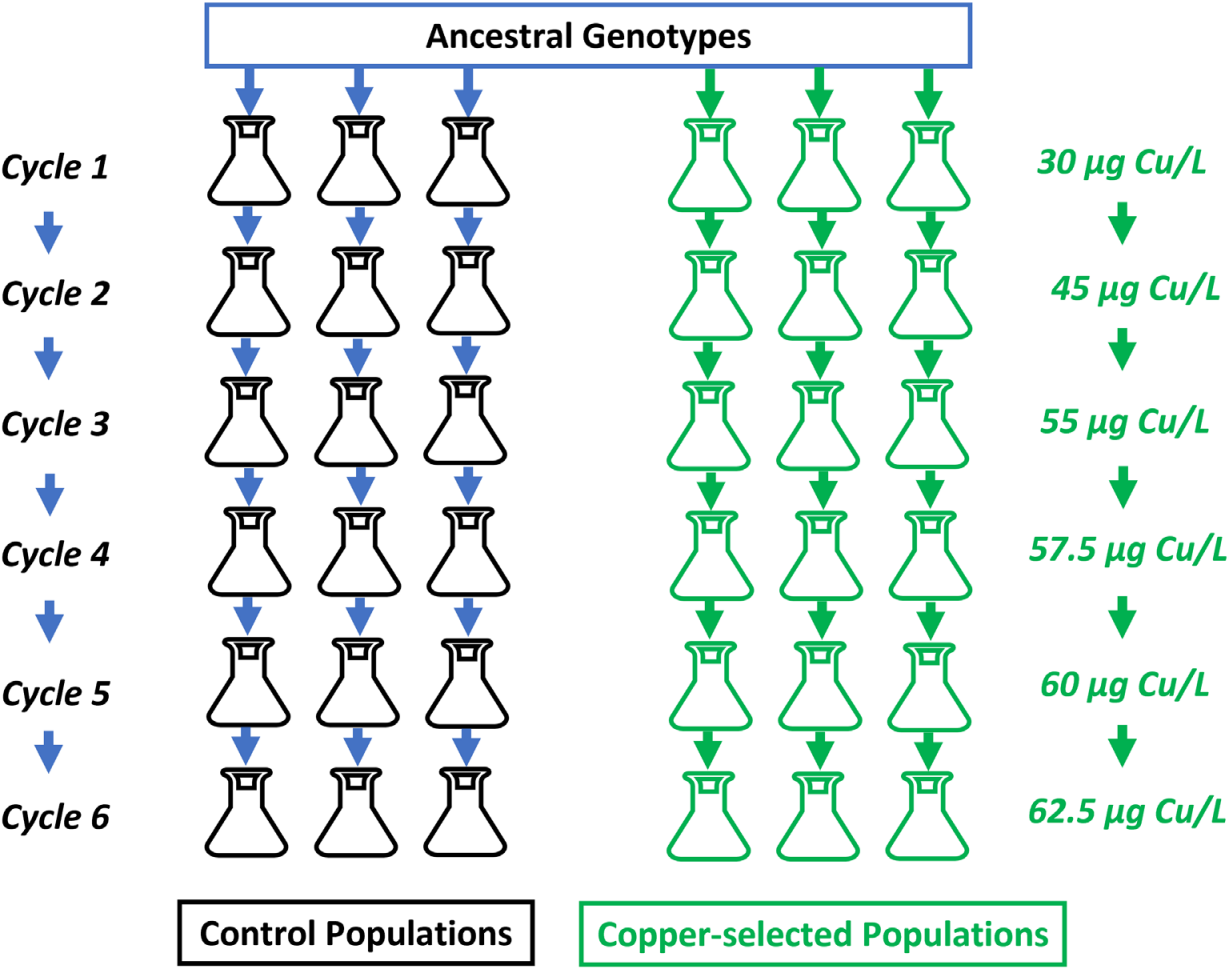
Design of the selection experiment. Using 50 randomly selected genotypes from an ancestral population, we created six genetically identical populations, three of which subsequently received stepwise increases in copper concentrations (starting at 30 μg Cu L^−1^ and finishing at 62.5 μg Cu L^−1^; ‘Cu‐selected populations’), whereas the other received no copper (‘Control populations’). During the process, all populations went through six subsequent cycles with each cycle consisting of a period of population growth followed by sexual reproduction (see Figure S1). At the beginning of a new cycle, each population was restarted from 50 clonal lines established from sexually produced dormant propagules from the previous cycle and the copper concentration in the Cu‐selected populations was augmented.

The selection experiment was designed to mimic the micro‐evolutionary response of natural populations to gradually increasing stress levels (Figure S1). Temperate populations of *B. calyciflorus* go through a cycle each growing season, during which a prolonged phase of clonal reproduction is followed by a bout of sexual reproduction that results in the formation of dormant propagules. After hatching, these propagules give rise to new genotypes that re‐establish the population at the beginning of a new growing season. To start, we created six laboratory populations composed of the same set of 50 clones. During the selection experiment, these populations all went simultaneously through six consecutive cycles (Figure 2). During the course of each such cycle, the population grew clonally until they reached densities high enough to induce sexual reproduction (i.e. ‘mixis’) (Serra et al., 2019). At the end of each cycle, dormant propagules were collected and used to establish new, genetically unique clonal lines. A new cycle was started by re‐initiating each population starting from a randomly selected set of 50 clones produced during the previous cycle. The remaining dormant propagules were stored at 4°C for future use (see Section 2.2). During the six cycles, three of the replicate populations were cultured under control conditions (‘Control populations’, no addition of copper). The remaining three replicate populations were reared under the same conditions but exposed to copper (‘Cu‐selected populations’). Between cycles, the copper concentration in the populations of the copper addition treatment was increased in steps (from 30, 45, 55, 57.5, 60 to 62.5 μg L^−1^ Cu in Cycles 1–6, respectively). During the selection experiment, populations were grown with the green alga *Chlamydomonas reinhardtii* in WC medium at a concentration of 1000 μmol C L^−1^ under constant light and a temperature of 22°C. Cultures in 500 mL flasks were continuously shaken on a rotating plate with 80 rotations min^−1^. The food suspension in the cultures was daily renewed.

### 2.2 Common garden transplant experiment

**FIGURE 3:**
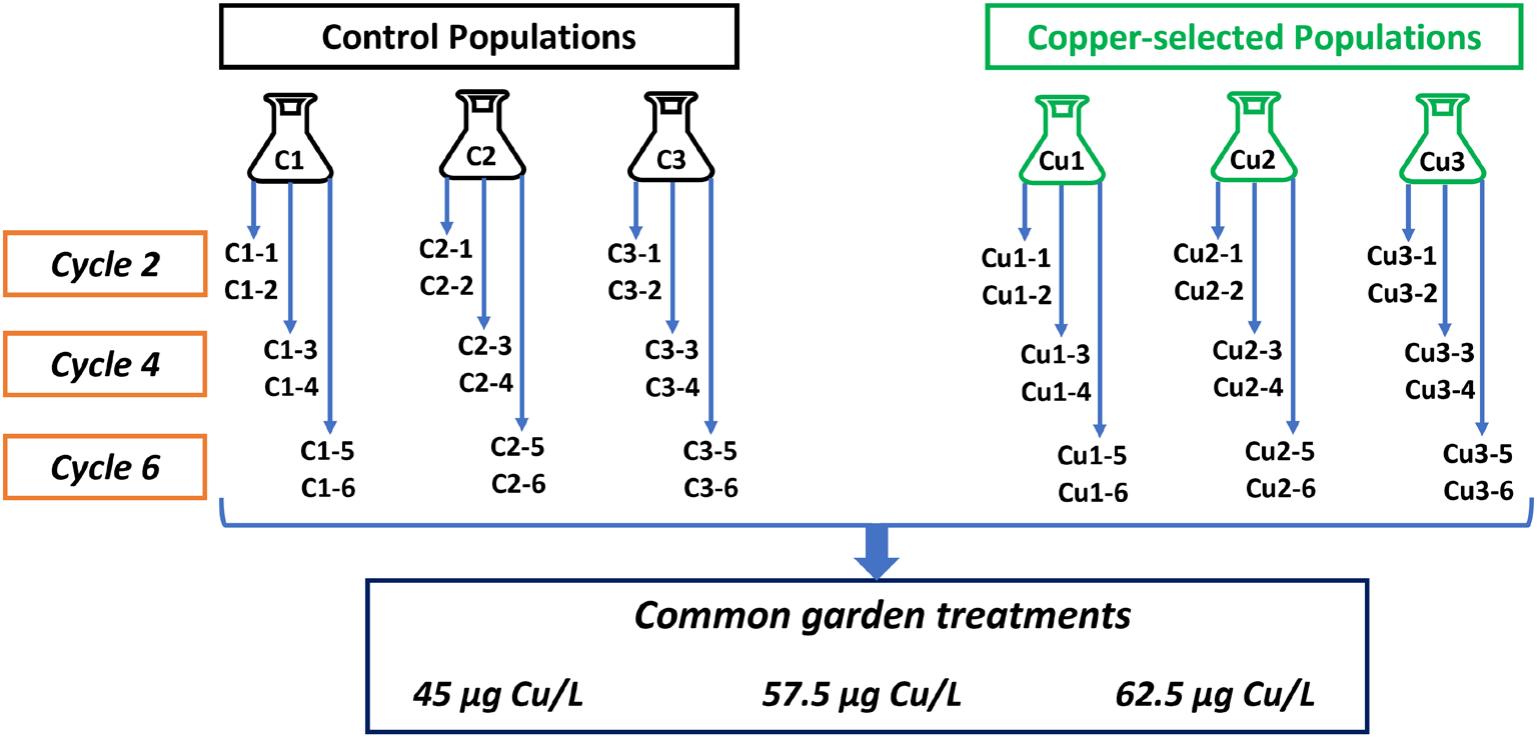
Design of common garden experiment. For each of the six populations obtained from the selection experiment, we established two clonal lines from dormant propagules produced during the second, fourth and sixth cycles. Experimental populations of all of these lines were exposed to copper addition treatments equal to those imposed during these cycles (i.e. 45, 57.5 and 62.5 μg L^−1^ Cu).

From each of the six populations in the selection experiment, two clonal lines were established from dormant propagules at three time points: Cycles 2, 4 and 6 (Figure 3). These clonal lines were then exposed to copper concentrations corresponding to each of these cycles (Cu45, Cu57.5 and Cu62.5 μg L^−1^) in a common garden experiment. The common garden experiment thus included 108 experimental units: three replicate populations from two selection histories (Control vs. Cu‐selected), with each population represented by six clones (two from each cycle), all exposed to the three copper treatments.

Before starting the common garden experiment, dormant propagules were hatched, and clonal populations were initiated and established under food‐satiating conditions (*C. reinhardtii*; 1000 μmol C L^−1^) in the absence of copper. After an initial phase of growing the populations, we created experimental units by transferring 16 randomly selected individuals to wells with 8 mL food suspension (*C. reinhardtii*; 1000 μmol C L^−1^). Cultures were maintained by daily transferring 16 random individuals to a fresh food suspension. Before the actual experiment, to acclimate populations, we gradually increased copper concentrations in the copper addition treatments until the final experimental target concentrations were reached. Subsequently, we monitored populations for a period of 5 days to ensure that daily population growth had stabilized. Data were collected by monitoring the populations during an additional 5 days. Every 24 h, just before transferring 16 individuals to fresh medium, we recorded the number of females, dead rotifers and loose dormant propagules. We made a distinction between females with no eggs, parthenogenetic eggs, non‐fertilized sexual eggs and dormant propagules. We also enumerated the number of eggs carried by fecund parthenogenetic females.

### 2.3 Copper determination experiment

To assess the actual Cu concentrations to which the rotifer populations were exposed during the common garden experiment, we conducted an additional experiment in which we cultured and monitored rotifer populations exposed to the Cu treatments (45, 57.5 and 62.5 μg L^−1^ Cu at 22°C, three replicates per treatment) in exactly the same way as in the common garden experiment. After the daily transfer of rotifers, the remaining medium of each unit (8 mL) was collected, filtered through a glass fibre filter and stored at −20°C during a period of 7 days. After the experiment, samples were pooled and Cu concentrations analysed with ICP‐MS by the Soil Chemistry Laboratory of Wageningen University and Research (WUR, Wageningen, The Netherlands). See Supporting Information for more details (Supplementary Methods 1).

### 2.4 Data analysis

Daily population growth was calculated as *r* = (lnN_t1_ − lnN_t0_)/*t*, where *r* is the exponential population growth rate, N_t0_ and N_t1_ are population sizes at the start and end of a 24‐h time interval, and *t* is the length of the time interval (i.e. 1 day). Mortality was calculated as the percentage of females that had died by the end of each time interval. Mean fecundity was calculated as the mean number of eggs per fecund parthenogenetic female. We also calculated the percentage of fecund females with sexual eggs (i.e. unfertilized sexual eggs and fertilized dormant propagules).

All endpoint variables were analysed with mixed effects models using R (v 4.3.1; R Core Team, 2023), with common garden treatment, selection history (i.e. Cu treatment in the selection experiment) and cycle (i.e. the cycle in the selection experiment after which clones were extracted) as fixed factors and clone as a random factor. Population growth and mean fecundity were fitted with linear mixed effect models (using REML and nloptwrap optimizer). The frequency of dead individuals and sexual eggs was analysed with generalized mixed effect models, using a binomial distribution and logit link function. For each of the four variables, the model with the most parsimonious multifactorial combination was selected based on AICc (Burnham & Anderson, 2004) using the ‘aictab’ function of the AICcmodavg package (v 2.3.2; Mazerolle, 2023). Models were constructed using the lmer package (v 1.1.34; Bates et al., 2014). Type III anova on linear mixed models was performed with the ‘anova’ function of the lmerTest package using Satterthwaite's degrees of freedom (v 3.1.3; Kuznetsova et al., 2017). Generalized mixed effect models were analysed with Type III Wald chi‐squared tests using the ‘Anova’ function of the car package (v 3.1.2; Fox & Weisberg, 2019). To evaluate differences among the cross‐factorial treatment levels, we applied a table‐wide Tukey post hoc comparison using the ‘glht’ and ‘cld’ functions of the multcomp package (1.4.25; Hothorn et al., 2008).

## 3 RESULTS

**FIGURE 4:**
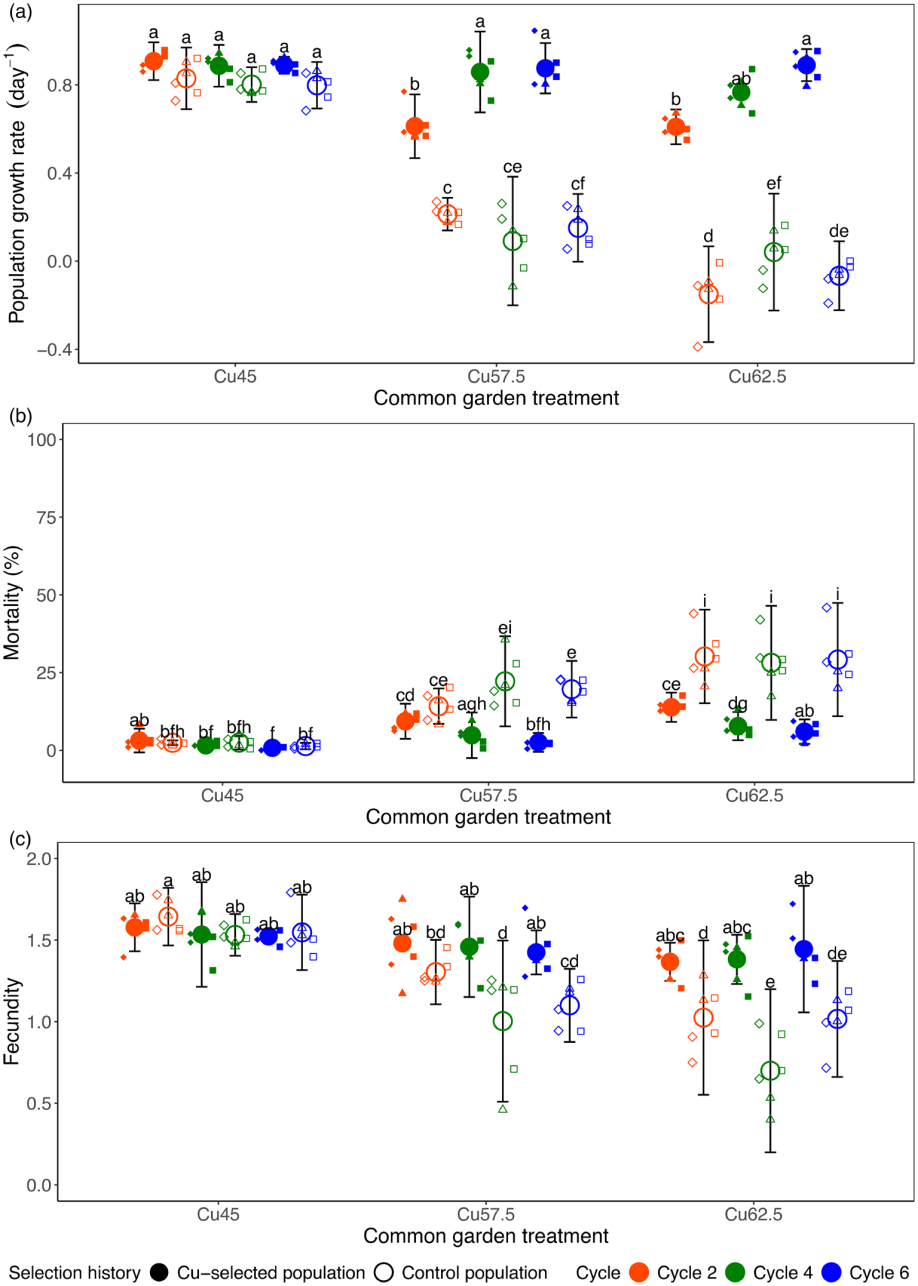
(a) Population growth rate (*r*) and (b) mortality and (c) fecundity of Control and Cu‐selected populations in response to the copper addition treatments of the common garden experiment. Selection history: Cu exposure history in the selection experiment; Cycle: Cycle after which clones were extracted from the populations in the selection experiment. Symbols and error bars represent means and 95% confidence intervals across population replicates of the selection experiment. Letters denote differences according to post hoc Tuckey pairwise comparisons (*α* = 0.05). Small symbols represent individual clones where different symbol types (squares, triangles and diamonds) identify pairs of clones that originated from the same population in the selection experiment (see also Figure 3).

Population growth rate *r* was impacted by a three‐way interaction between common garden Cu addition, population selection history and the cycle of isolation (Tables S1 and S2; Figure 4A). In the lowest Cu treatment level (Cu45), population growth rates tended to be higher in Cu‐selected than Control populations, although these differences were not found to be significant according to Tukey post hoc tests. However, growth rates of all Control populations decreased strongly with increasing copper levels (Cu57.5 and Cu62.5 treatments) and approximated zero or became negative at the highest copper treatment. In contrast, the response of Cu‐selected populations to Cu addition was much less pronounced and depended strongly on cycle, that is, the maximum copper concentration they had been exposed to during the selection experiment. When exposed to intermediate and very high Cu concentrations (Cu57.5 and Cu62.5), Cu‐selected populations isolated after the second cycle, that is, after exposure to 45 μg Cu L^−1^ (SelHist_Cu45) showed a reduced performance compared to populations isolated during the fourth and sixth cycles (SelHist_Cu57.5 and SelHist_Cu62.5). Yet, SelHist_Cu45 populations demonstrated a much higher ability to cope with high (Cu57.5) and very high (Cu62.5) copper concentrations than any of the Control populations. While the growth rates of SelHist_Cu45 populations were 20%–30% lower than the SelHist_Cu57.5 and SelHist_Cu62.5 populations when exposed to intermediate and high Cu concentrations, the Control populations declined by 65% and >95%, respectively (Figure 4A).

For mortality, the generalized mixed model with the lowest AIC included a three‐way interaction between common garden treatments, population selection history and the cycle of isolation (Tables S1 and S3; Figure 4B). Differences in mortality between the clonal populations exposed to the different Cu treatments were found to largely mirror those of the population growth rate. In the low copper treatment (Cu45), mortality was very low, and no significant differences were found between Cu‐selected and Control populations. Mortality increased with increasing copper concentrations, although this effect was more pronounced for the control (mean: 30%) than Cu‐selected populations (mean: 10%). When exposed to intermediate and very high Cu concentrations (Cu57.5 and Cu62.5), SelHist_Cu45 populations showed higher mortality than populations adapted to higher Cu concentrations (SelHist_Cu57.5 and SelHist_Cu62.5). Nevertheless, SelHist_Cu45 populations demonstrated much lower mortality under high (Cu62.5) copper concentrations than Control populations (Figure 4B).

Fecundity was determined by a significant interaction between common garden treatments and selection history (Tables S1 and S2; Figure 4C). In populations of both selection histories, fecundity decreased with increasing copper concentrations, but this trend was more pronounced in the Control than in the Cu‐selected populations (Figure 4C).

The most parsimonious model for the fraction of females with sexual eggs consisted of a two‐way interaction between common garden treatments and population selection history (Tables S1 and S3; Figure S2). The effect of the moment of clone isolation was not significant. In contrast to Cu‐selected populations, Control populations tended to respond to an increased Cu concentration with a reduction of females with sexual eggs in the high Cu treatment (Cu62.5) (Figure S2), although this pattern was not shown to be significant in the Tukey post hoc comparison.

Mean Cu45, Cu57.5 and Cu62.5 treatments corresponded to actually measured concentrations of 44, 52 and 54 μg L^−1^, respectively (Figure S3).

## 4 DISCUSSION

In the common garden experiment, rotifer populations that had been exposed to Cu during the selection experiment showed a much higher ability to tolerate Cu than populations without a history of exposure to Cu (control populations). Indeed, control populations suffered a strong reduction in their population growth when exposed to intermediate (57.5 μg L^−1^) and high (62.5 μg L^−1^) Cu levels, while no such reduction was observed in populations with a history of selection by intermediate and high Cu concentrations. These performance differences were found to be driven by higher mortality rates and reduced fecundities in the control populations.

With regard to our initial research question, it is of particular interest to compare the response of populations adapted to low Cu (45 μg L^−1^; Cycle 2) with the control populations. These low Cu‐adapted populations barely outperformed the control populations when exposed to concentrations equal to those they had been exposed to in the selection experiment (Figure 4A). In contrast, they performed much better than the control populations when exposed to intermediate and high Cu treatments, mainly as the result of lower mortality rates. Adaptation of populations to selection pressure exerted by low levels of Cu thus yielded a fitness advantage that became especially pronounced at much higher Cu concentrations. This indicates that adaptation to low Cu levels involves modifications in stress coping mechanisms that are also highly effective in dealing with much higher levels of this stressor. Our results thus provide substantial support for the idea of micro‐evolutionary priming in animals, where adaptation to (very) low stress levels renders populations disproportionately robust to the negative effects of future acute stress events.

Differences in exposure duration may have influenced common garden performance of populations isolated during the various cycles of the selection experiment. Indeed, populations from which clones were isolated during Cycle 2 not only experienced selection by lower copper concentrations, exposure to copper also lasted during fewer generations compared to populations at later cycles. However, this affects only the comparison between populations of different cycles and does not alter the key finding that Cycle 2 populations strongly outperformed control populations in treatments with high copper concentrations. On the contrary, the observation that priming occurred despite the shorter exposure to low‐stress conditions underscores the significant impact of selection under low‐stress levels on tolerance.

Different strategies exist by which organisms cope with a fluctuating environment (Bernhardt et al., 2020). These strategies vary depending on the degree of predictability of the environment and the time scale over which the environment varies compared to the generation time of the organisms. When fluctuations are unpredictable and occur on an intergenerational time scale, populations may evolve bet‐hedging strategies (Childs et al., 2010). In more predictable environments, organisms may use environmental cues to anticipate a changing environment through phenotypic plasticity, within their own life cycle, or by inducing their offspring. These strategies are typically shaped as a result of an evolutionary response to a specific history of temporally varying selection regimes. This, by definition, is in stark contrast to the micro‐evolutionary priming described here, where populations are able to cope with acute stress without having been exposed to such high stress before. Because our common garden experiments involved constant Cu levels, we should be prudent in making statements about how the performance of evolved rotifer populations would differ from control populations in more dynamic environments. Nevertheless, our results strongly suggest that low Cu‐adapted populations should be better able to cope with an environment characterized by strong fluctuations of Cu than naïve populations. Given that low Cu‐adapted populations have never been exposed to acute Cu stress in their (recent) evolutionary history, this coping ability cannot have resulted from adaptation to such high concentrations. Instead, it should be regarded as a byproduct of adaptation to low Cu concentrations.

Micro‐evolutionary priming may have important implications for the long‐term persistence of populations that face anthropogenic change (Gonzalez & Bell, 2013). For many stressors to natural systems, a gradual increase in the long‐term mean is often accompanied by an increase in the height of incidental peak values (Figure 1a; Bernhardt et al., 2020). For example, in the case of metal contamination, the intensity of toxic metal stress at a site can vary greatly with time because bioavailability and toxicity are determined by temporal variation in pollution loads and the chemical and physical environment (e.g. pH, dissolved organic carbon, oxygen levels; Mbandzi et al., 2022). Sediments, for example, act as important sinks for metals. However, the resuspension of contaminated sediments, due to disturbance events caused by natural (e.g. winds, bioturbation, storm surge or precipitation) or anthropogenic origin (e.g. dredging, shipping, trawling), may turn them into an important source of bioavailable metals, especially under conditions that promote their dissociation and remobilization (do Nascimento Monte et al., 2021; Roberts, 2012). The gradual accumulation of metals in an ecosystem over time is therefore associated with a strongly increased risk for acute peaks of metal stress (Birch & O'Hea, 2007), risks that may be exacerbated by the increase in the frequency of wind storms, heavy rainfall and floods associated with climate change (Birch & O'Hea, 2007; Roberts, 2012). Although populations would be expected to be imperilled at the occasion of such peaks, our results suggest that initial micro‐evolutionary adaptation to low levels of a metal stressor may strongly contribute to their persistence in the face of such peak events. Although micro‐evolutionary priming has received no or very little attention, it may be an important determinant of the persistence of populations that face an increased risk of acute stress due to other types of stressors, such as other toxic contaminants, salts or heatwaves as well (Brans et al., 2017; Coldsnow et al., 2017; Khan et al., 2011; Weston et al., 2013).

Micro‐evolutionary priming may not only have a major impact on the robustness of populations under high temporal stress variability but could also potentially influence the spatial dynamics of metapopulations and, by extension, metacommunities (De Meester et al., 2011; Leibold et al., 2022). Particularly in landscapes with high spatial heterogeneity in stress levels, we expect that micro‐evolutionary priming may contribute to the spatial expansion of metapopulations because it may increase the ability of populations from stress‐free sites to colonize high‐stress sites (Figure 1b). More specifically, in a landscape, low‐stress sites could serve as stepping stones to high‐stress sites for naïve populations from stress‐free sites. This is because they provide colonizers of stress‐free sites the opportunity to locally adapt to low‐stress conditions. These populations may then become equipped to colonize high‐stress sites that would otherwise remain inaccessible for genotypes originating from stress free environments.

In ecotoxicology, much work has focused on the question of how multigenerational exposure of organisms to toxins affects their tolerance. The tolerance response of organisms such as *Daphnia*, *Gammarus* and copepods to multigenerational exposure to metals has been shown to exhibit considerable variability between studies. Depending on the test organism, metal identity, metal concentration and food conditions, multigenerational exposure has been found to lead to reduced tolerance (Araujo et al., 2019; Guan & Wang, 2006; Li et al., 2015; Völker et al., 2013), whereas in other cases, it has resulted in increased tolerance (Kwok et al., 2009; Shaw et al., 2019; Sun et al., 2014). Current evidence supporting epigenetics‐mediated transgenerational hormetic responses, where exposure to metal toxicity in one generation enhances subsequent generations' tolerance to higher concentrations than initially encountered (Agathokleous et al., 2021; Sebastiano et al., 2022), remains limited and extends only over a few generations (Bossuyt & Janssen, 2004; Muyssen & Janssen, 2004). Studies typically document responses of successive generations descended from single clonally reproducing genotypes or pairs of sexually reproducing individuals. As a result, they rule out population‐level micro‐evolutionary adaptation. In contrast, our selection experiment was initiated with multiclonal populations that underwent repeated bouts of sexual reproduction during the stepwise increase in stressor intensity over the course of the experiment. Given that rotifer populations are known to harbour substantial interclonal variation in their ability to cope with a variety of stressors (Alcántara Rodríguez et al., 2012; Lemmen et al., 2022; Walczyńska et al., 2017; Xi et al., 2019; Zhu et al., 2022), including metal stress (Orr et al., 2022), the adaptation observed in our study most likely occurred mainly through directional selection on favourable allele combinations formed after each bout of sexual reproduction. In the common garden experiment, genotypes from populations with a history of exposure to low Cu concentrations showed strong differences from control populations in the extent to which they were able to cope with high Cu levels, despite having been exposed to the same conditions for many generations during the acclimation period prior to the common garden experiment. For this reason, the observed divergence in fitness response between these populations can be more easily explained as the consequence of micro‐evolutionary adaptation than by epigenetics‐mediated transgenerational hormetic responses.

Micro‐evolutionary priming is likely to have different and more long‐lasting effects on populations than transgenerational hormetic responses (Kristensen et al., 2020). Although some studies have provided evidence for transgenerational hormetic responses to last for many generations (Shi et al., 2021; Yue et al., 2021), most evidence for this mechanism is still limited to the F1 or F2 generation (Margus et al., 2019; Okabe et al., 2021; Tsui & Wang, 2005; Ullah et al., 2020). In contrast, the impact of micro‐evolutionary priming is expected to be more durable over time. Due to its genetic basis, tolerance to acute concentrations may be transferred across numerous generations and bridge long periods of very low to negligible stressor levels. The period for which such an adaptation will be maintained in a population will obviously depend greatly on whether there are fitness costs involved (Vigneron et al., 2015) and the frequency of exposure to the stressor.

## 5 CONCLUSIONS

Consideration of adaptive processes is essential for a better understanding and prediction of how animal populations will respond to anthropogenic change. Our study provides evidence that a multigenerational exposure of genetically diverse rotifer populations to low levels of Cu stress allows these populations to evolve tolerance for much higher levels of this stress. Our results point to a potentially very important mechanism that has hitherto received very limited attention in animal populations. Further research is needed with other taxonomic groups and different types of stressors to confirm the generality of such ‘micro‐evolutionary priming’. This is especially relevant given the general observation that the gradual increase of stressors tends to be associated with more extreme stress peaks in natural systems. Evaluating the extent to which organisms can increase their coping abilities to acute stress by micro‐evolutionary adaptation to relatively low‐stress levels is important for assessing the potential response of (meta)populations, communities and associated ecosystem functions in a rapidly changing world.

## AUTHOR CONTRIBUTIONS

Steven A. J. Declerck and Shuwen Han conceived the ideas and designed the experiment. Shuwen Han executed the experiment and collected the data. Shuwen Han and Steven A. J. Declerck analysed the data. Shuwen Han, Steven A. J. Declerck and Paul J. Van den Brink interpreted the data and all contributed to writing the manuscript. All authors contributed critically to the drafts and gave final approval for publication.

## CONFLICT OF INTEREST STATEMENT

The authors declare no conflicts of interest.

## STATEMENT ON INCLUSION

Our study brings together authors from different countries. All authors were engaged early on with the research and study design to ensure that the diverse sets of perspectives they represent were considered from the onset.

## Supporting information


**Figure S1.** The selection experiment was started with creating 6 populations consisting of an identical set of 50 clones.
**Figure S2.** Percentage of females with sexual eggs in Control and Cu selected populations in response to the copper addition treatments of the common garden experiment.
**Figure S3.** Copper concentration in the medium for rotifer populations in response to copper addition treatments.
**Table S1.** Model selection based on AICc values.
**Table S2.** General linear mixed effects models for population growth rate and fecundity.
**Table S3.** Generalized linear mixed effects models (binomial distribution with log‐link function) for mortality (counts of dead versus alive rotifers) and sexual investment (counts of females with sexual eggs versus parthenogenetic eggs).
**Supplementary Methods 1.** Methodological description of the Copper Concentration Assessment.

## Data Availability

Data available from the Dryad Digital Repository https://doi.org/10.5061/dryad.z34tmpgng (Han et al., 2025).

## References

[jane70012-bib-0001] Agathokleous, E. , Brown, P. H. , & Calabrese, E. J. (2021). A gift from parent to offspring: Transgenerational hormesis. Trends in Plant Science, 26(11), 1098–1100. 10.1016/j.tplants.2021.08.006 34507888

[jane70012-bib-0002] Agathokleous, E. , & Calabrese, E. J. (2020). Environmental toxicology and ecotoxicology: How clean is clean? Rethinking dose‐response analysis. Science of the Total Environment, 746, 138769. 10.1016/j.scitotenv.2020.138769 32389333

[jane70012-bib-0003] Agathokleous, E. , Guedes, R. N. C. , Calabrese, E. J. , Fotopoulos, V. , & Azevedo, R. A. (2022). Transgenerational hormesis: What do parents sacrifice for their offspring? Current Opinion in Environmental Science & Health, 29, 100380. 10.1016/j.coesh.2022.100380

[jane70012-bib-0004] Alcántara Rodríguez, J. A. , Ciros Pérez, J. , Ortega Mayagoitia, E. , Serrania Soto, C. R. , & Piedra Ibarra, E. (2012). Local adaptation in populations of a Brachionus group plicatilis cryptic species inhabiting three deep crater lakes in Central Mexico. Freshwater Biology, 57(4), 728–740. 10.1111/j.1365-2427.2012.02738.x

[jane70012-bib-0005] Araujo, G. S. , Abessa, D. M. S. , Soares, A. M. V. M. , & Loureiro, S. (2019). Multi‐generational exposure to Pb in two monophyletic Daphnia species: Individual, functional and population related endpoints. Ecotoxicology and Environmental Safety, 173, 77–85. 10.1016/j.ecoenv.2019.02.001 30769206

[jane70012-bib-0006] Bates, D. , Mächler, M. , Bolker, B. , & Walker, S. (2014). Fitting linear mixed‐effects models using lme4 . (arXiv:1406.5823). arXiv. http://arxiv.org/abs/1406.5823

[jane70012-bib-0007] Bergland, A. O. , Behrman, E. L. , O'Brien, K. R. , Schmidt, P. S. , & Petrov, D. A. (2014). Genomic evidence of rapid and stable adaptive oscillations over seasonal time scales in Drosophila. PLoS Genetics, 10(11), e1004775. 10.1371/journal.pgen.1004775 25375361 PMC4222749

[jane70012-bib-0008] Bernhardt, J. R. , O'Connor, M. I. , Sunday, J. M. , & Gonzalez, A. (2020). Life in fluctuating environments. Philosophical Transactions of the Royal Society, B: Biological Sciences, 375(1814), 20190454. 10.1098/rstb.2019.0454 PMC7662201 33131443

[jane70012-bib-0009] Birch, G. , & O'Hea, L. (2007). The chemistry of suspended particulate material in a highly contaminated embayment of Port Jackson (Australia) under quiescent, high‐wind and heavy‐rainfall conditions. Environmental Geology, 53(3), 501–516. 10.1007/s00254-007-0662-5

[jane70012-bib-0010] Bossuyt, B. T. A. , & Janssen, C. R. (2004). Influence of multigeneration acclimation to copper on tolerance, energy reserves, and homeostasis of Daphnia magna Straus. Environmental Toxicology and Chemistry, 23(8), 2029–2037. 10.1897/03-377 15352494

[jane70012-bib-0011] Brans, K. I. , Jansen, M. , Vanoverbeke, J. , Tüzün, N. , Stoks, R. , & Meester, L. D. (2017). The heat is on: Genetic adaptation to urbanization mediated by thermal tolerance and body size. Global Change Biology, 23(12), 5218–5227. 10.1111/gcb.13784 28614592

[jane70012-bib-0012] Brevik, K. , Lindström, L. , McKay, S. D. , & Chen, Y. H. (2018). Transgenerational effects of insecticides—Implications for rapid pest evolution in agroecosystems. Current Opinion in Insect Science, 26, 34–40. 10.1016/j.cois.2017.12.007 29764658

[jane70012-bib-0013] Burnham, K. P. , & Anderson, D. R. (2004). Multimodel inference: Understanding AIC and BIC in model selection. Sociological Methods & Research, 33(2), 261–304. 10.1177/0049124104268644

[jane70012-bib-0014] Childs, D. Z. , Metcalf, C. J. E. , & Rees, M. (2010). Evolutionary bet‐hedging in the real world: Empirical evidence and challenges revealed by plants. Proceedings of the Royal Society B: Biological Sciences, 277(1697), 3055–3064. 10.1098/rspb.2010.0707 PMC2982066 20573624

[jane70012-bib-0015] Coldsnow, K. D. , Mattes, B. M. , Hintz, W. D. , & Relyea, R. A. (2017). Rapid evolution of tolerance to road salt in zooplankton. Environmental Pollution, 222, 367–373. 10.1016/j.envpol.2016.12.024 28065573

[jane70012-bib-0016] Costantini, D. , Metcalfe, N. B. , & Monaghan, P. (2010). Ecological processes in a hormetic framework. Ecology Letters, 13(11), 1435–1447. 10.1111/j.1461-0248.2010.01531.x 20849442

[jane70012-bib-0017] De Meester, L. , Van Doorslaer, W. , Geerts, A. , Orsini, L. , & Stoks, R. (2011). Thermal genetic adaptation in the water flea daphnia and its impact: An evolving metacommunity approach. Integrative and Comparative Biology, 51(5), 703–718. 10.1093/icb/icr027 21775388

[jane70012-bib-0018] de Villemereuil, P. , Charmantier, A. , Arlt, D. , Bize, P. , Brekke, P. , Brouwer, L. , Cockburn, A. , Côté, S. D. , Dobson, F. S. , Evans, S. R. , Festa‐Bianchet, M. , Gamelon, M. , Hamel, S. , Hegelbach, J. , Jerstad, K. , Kempenaers, B. , Kruuk, L. E. B. , Kumpula, J. , Kvalnes, T. , & Chevin, L.‐M. (2020). Fluctuating optimum and temporally variable selection on breeding date in birds and mammals. Proceedings of the National Academy of Sciences of the United States of America, 117(50), 31969–31978. 10.1073/pnas.2009003117 33257553 PMC7116484

[jane70012-bib-0019] Declerck, S. A. J. , Malo, A. R. , Diehl, S. , Waasdorp, D. , Lemmen, K. D. , Proios, K. , & Papakostas, S. (2015). Rapid adaptation of herbivore consumers to nutrient limitation: Eco‐evolutionary feedbacks to population demography and resource control. Ecology Letters, 18(6), 553–562. 10.1111/ele.12436 25913306

[jane70012-bib-0020] Declerck, S. A. J. , & Papakostas, S. (2017). Monogonont rotifers as model systems for the study of micro‐evolutionary adaptation and its eco‐evolutionary implications. Hydrobiologia, 796(1), 131–144. 10.1007/s10750-016-2782-y

[jane70012-bib-0021] Derry, A. M. , Fraser, D. J. , Brady, S. P. , Astorg, L. , Lawrence, E. R. , Martin, G. K. , Matte, J.‐M. , Negrín Dastis, J. O. , Paccard, A. , Barrett, R. D. H. , Chapman, L. J. , Lane, J. E. , Ballas, C. G. , Close, M. , & Crispo, E. (2019). Conservation through the lens of (mal)adaptation: Concepts and meta‐analysis. Evolutionary Applications, 12(7), 1287–1304. 10.1111/eva.12791 31417615 PMC6691223

[jane70012-bib-0022] Diamond, S. E. , & Chick, L. D. (2018). The Janus of macrophysiology: Stronger effects of evolutionary history, but weaker effects of climate on upper thermal limits are reversed for lower thermal limits in ants. Current Zoology, 64(2), 223–230. 10.1093/cz/zox072 30402063 PMC5905527

[jane70012-bib-0023] do Nascimento Monte, C. , de Castro Rodrigues, A. P. , de Freitas, A. R. , Braz, B. F. , Freire, A. S. , Cordeiro, R. C. , Santelli, R. E. , & Machado, W. T. V. (2021). Ecological risks associated to trace metals of contaminated sediments from a densely urbanized tropical eutrophic estuary. Environmental Monitoring and Assessment, 193(12), 1–15. 10.1007/s10661-021-09552-7 34731306

[jane70012-bib-0024] Doorslaer, W. V. , Vanoverbeke, J. , Duvivier, C. , Rousseaux, S. , Jansen, M. , Jansen, B. , Feuchtmayr, H. , Atkinson, D. , Moss, B. , Stoks, R. , & Meester, L. D. (2009). Local adaptation to higher temperatures reduces immigration success of genotypes from a warmer region in the water flea Daphnia. Global Change Biology, 15(12), 3046–3055. 10.1111/j.1365-2486.2009.01980.x

[jane70012-bib-0025] Easterling, D. R. , Meehl, G. A. , Parmesan, C. , Changnon, S. A. , Karl, T. R. , & Mearns, L. O. (2000). Climate extremes: Observations, modeling, and impacts. Science, 289(5487), 2068–2074. 10.1126/science.289.5487.2068 11000103

[jane70012-bib-0026] Fox, J. , & Weisberg, S. (2019). Nonlinear regression, nonlinear least squares, and nonlinear mixed models in R. Population, 150, 200.

[jane70012-bib-0027] Geerts, A. N. , Vanoverbeke, J. , Vanschoenwinkel, B. , Van Doorslaer, W. , Feuchtmayr, H. , Atkinson, D. , Moss, B. , Davidson, T. A. , Sayer, C. D. , & De Meester, L. (2015). Rapid evolution of thermal tolerance in the water flea daphnia. Nature Climate Change, 5(7), 665–668. 10.1038/nclimate2628

[jane70012-bib-0028] Gonzalez, A. , & Bell, G. (2013). Evolutionary rescue and adaptation to abrupt environmental change depends upon the history of stress. Philosophical Transactions of the Royal Society, B: Biological Sciences, 368(1610), 20120079. 10.1098/rstb.2012.0079 PMC3538446 23209161

[jane70012-bib-0029] Guan, R. , & Wang, W.‐X. (2006). Multigenerational cadmium acclimation and biokinetics in Daphnia magna. Environmental Pollution, 141(2), 343–352. 10.1016/j.envpol.2005.08.036 16202491

[jane70012-bib-0030] Gullberg, E. , Cao, S. , Berg, O. G. , Ilbäck, C. , Sandegren, L. , Hughes, D. , & Andersson, D. I. (2011). Selection of resistant bacteria at very low antibiotic concentrations. PLoS Pathogens, 7(7), e1002158. 10.1371/journal.ppat.1002158 21811410 PMC3141051

[jane70012-bib-0031] Han, S. , Van den Brink, P. J. , & Declerck, S. A. J. (2025). Data from: Adapting to an increasingly stressful environment: Experimental evidence for ‘micro‐evolutionary priming’. *Dryad Digital Repository*. 10.5061/dryad.z34tmpgng PMC12056352 39972545

[jane70012-bib-0032] Hothorn, T. , Bretz, F. , & Westfall, P. (2008). Simultaneous inference in general parametric models. Biometrical Journal, 50(3), 346–363. 10.1002/bimj.200810425 18481363

[jane70012-bib-0033] Khan, F. R. , Irving, J. R. , Bury, N. R. , & Hogstrand, C. (2011). Differential tolerance of two *Gammarus pulex* populations transplanted from different metallogenic regions to a polymetal gradient. Aquatic Toxicology, 102(1), 95–103. 10.1016/j.aquatox.2011.01.001 21371617

[jane70012-bib-0034] King, J. G. , & Hadfield, J. D. (2019). The evolution of phenotypic plasticity when environments fluctuate in time and space. Evolution Letters, 3(1), 15–27. 10.1002/evl3.100 30788139 PMC6369965

[jane70012-bib-0035] Kristensen, T. N. , Ketola, T. , & Kronholm, I. (2020). Adaptation to environmental stress at different timescales. Annals of the New York Academy of Sciences, 1476(1), 5–22. 10.1111/nyas.13974 30259990

[jane70012-bib-0036] Kuznetsova, A. , Brockhoff, P. B. , & Christensen, R. H. B. (2017). lmerTest package: Tests in linear mixed effects models. Journal of Statistical Software, 82, 1–26. 10.18637/jss.v082.i13

[jane70012-bib-0037] Kwok, K. W. H. , Grist, E. P. M. , & Leung, K. M. Y. (2009). Acclimation effect and fitness cost of copper resistance in the marine copepod *Tigriopus japonicus* . Ecotoxicology and Environmental Safety, 72(2), 358–364. 10.1016/j.ecoenv.2008.03.014 18842299

[jane70012-bib-0038] Lagator, M. , Uecker, H. , & Neve, P. (2021). Adaptation at different points along antibiotic concentration gradients. Biology Letters, 17(5), 20200913. 10.1098/rsbl.2020.0913 33975485 PMC8113895

[jane70012-bib-0039] Leibold, M. A. , Govaert, L. , Loeuille, N. , De Meester, L. , & Urban, M. C. (2022). Evolution and community assembly across spatial scales. Annual Review of Ecology, Evolution, and Systematics, 53(1), 299–326. 10.1146/annurev-ecolsys-102220-024934

[jane70012-bib-0072] Lemmen, K. D. , Zhou, L. , Papakostas, S. , & Declerck, S. A. J. (2022). An experimental test of the growth rate hypothesis as a predictive framework for microevolutionary adaptation. Ecology, 104(1), e3853. 10.1002/ecy.3853 36054549 PMC10078216

[jane70012-bib-0041] Li, H. , Shi, L. , Wang, D. , & Wang, M. (2015). Impacts of mercury exposure on life history traits of *Tigriopus japonicus*: Multigeneration effects and recovery from pollution. Aquatic Toxicology, 166, 42–49. 10.1016/j.aquatox.2015.06.015 26210816

[jane70012-bib-0042] Li, X. (2020). Heat wave trends in Southeast Asia during 1979–2018: The impact of humidity. Science of the Total Environment, 721, 137664. 10.1016/j.scitotenv.2020.137664 32182463

[jane70012-bib-0043] Margus, A. , Piiroinen, S. , Lehmann, P. , Tikka, S. , Karvanen, J. , & Lindström, L. (2019). Sublethal pyrethroid insecticide exposure carries positive fitness effects over generations in a Pest insect. Scientific Reports, 9(1), Article 1. 10.1038/s41598-019-47473-1 PMC6683203 31383885

[jane70012-bib-0044] Mazerolle, M. J. (2023). AICcmodavg: Model selection and multimodel inference based on (Q)AIC(c). R package version 2.3.3. https://cran.r‐project.org/package=AICcmodavg

[jane70012-bib-0045] Mbandzi, N. , Vincent Nakin, M. D. , & Oyedeji, A. O. (2022). Temporal and spatial variation of heavy metal concentration in four limpet species along the southeast coast of South Africa. Environmental Pollution, 302, 119056. 10.1016/j.envpol.2022.119056 35227843

[jane70012-bib-0046] Meek, M. H. , Beever, E. A. , Barbosa, S. , Fitzpatrick, S. W. , Fletcher, N. K. , Mittan‐Moreau, C. S. , Reid, B. N. , Campbell‐Staton, S. C. , Green, N. F. , & Hellmann, J. J. (2023). Understanding local adaptation to prepare populations for climate change. Bioscience, 73(1), 36–47. 10.1093/biosci/biac101

[jane70012-bib-0047] Michaloudi, E. , Papakostas, S. , Stamou, G. , Neděla, V. , Tihlaříková, E. , Zhang, W. , & Declerck, S. A. J. (2018). Reverse taxonomy applied to the *Brachionus calyciflorus* cryptic species complex: Morphometric analysis confirms species delimitations revealed by molecular phylogenetic analysis and allows the (re)description of four species. PLoS One, 13(9), e0203168. 10.1371/journal.pone.0203168 30235243 PMC6147415

[jane70012-bib-0048] Muyssen, B. T. A. , & Janssen, C. R. (2004). Multi‐generation cadmium acclimation and tolerance in Daphnia magna Straus. Environmental Pollution, 130(3), 309–316. 10.1016/j.envpol.2004.01.003 15182964

[jane70012-bib-0049] Okabe, E. , Uno, M. , Kishimoto, S. , & Nishida, E. (2021). Intertissue small RNA communication mediates the acquisition and inheritance of hormesis in *Caenorhabditis elegans* . Communications Biology, 4(1), Article 1. 10.1038/s42003-021-01692-3 PMC7886853 33594200

[jane70012-bib-0050] Orr, J. A. , Luijckx, P. , Arnoldi, J.‐F. , Jackson, A. L. , & Piggott, J. J. (2022). Rapid evolution generates synergism between multiple stressors: Linking theory and an evolution experiment. Global Change Biology, 28(5), 1740–1752. 10.1111/gcb.15633 33829610

[jane70012-bib-0051] Pfenninger, M. , & Foucault, Q. (2022). Population genomic time series data of a natural population suggests adaptive tracking of fluctuating environmental changes. Integrative and Comparative Biology, 62(6), 1812–1826. 10.1093/icb/icac098 35762661

[jane70012-bib-0052] R Core Team . (2023). R: A Language and environment for statistical computing. R Foundation for Statistical Computing. https://www.R‐project.org/

[jane70012-bib-0053] Roberts, D. A. (2012). Causes and ecological effects of resuspended contaminated sediments (RCS) in marine environments. Environment International, 40, 230–243. 10.1016/j.envint.2011.11.013 22244126

[jane70012-bib-0054] Samani, P. , & Bell, G. (2010). Adaptation of experimental yeast populations to stressful conditions in relation to population size. Journal of Evolutionary Biology, 23(4), 791–796. 10.1111/j.1420-9101.2010.01945.x 20149025

[jane70012-bib-0055] Sandegren, L. (2014). Selection of antibiotic resistance at very low antibiotic concentrations. Upsala Journal of Medical Sciences, 119(2), 103–107. 10.3109/03009734.2014.904457 24694026 PMC4034545

[jane70012-bib-0056] Sebastiano, M. , Messina, S. , Marasco, V. , & Costantini, D. (2022). Hormesis in ecotoxicological studies: A critical evolutionary perspective. Current Opinion in Toxicology, 29, 25–30. 10.1016/j.cotox.2022.01.002

[jane70012-bib-0057] Serra, M. , García‐Roger, E. M. , Ortells, R. , & Carmona, M. J. (2019). Cyclically parthenogenetic rotifers and the theories of population and evolutionary ecology. Limnetica, 38(1), 67–93.

[jane70012-bib-0058] Shaw, J. R. , Colbourne, J. K. , Glaholt, S. P. , Turner, E. , Folt, C. L. , & Chen, C. Y. (2019). Dynamics of cadmium acclimation in *Daphnia pulex*: Linking fitness costs, cross‐tolerance, and hyper‐induction of metallothionein. Environmental Science & Technology, 53(24), 14670–14678. 10.1021/acs.est.9b05006 31738529

[jane70012-bib-0059] Shi, Y. , Meng, X. , & Zhang, J. (2021). Multi‐ and trans‐generational effects of N‐butylpyridium chloride on reproduction, lifespan, and pro/antioxidant status in *Caenorhabditis elegans* . Science of the Total Environment, 778, 146371. 10.1016/j.scitotenv.2021.146371 34030357

[jane70012-bib-0060] Sun, P. Y. , Foley, H. B. , Handschumacher, L. , Suzuki, A. , Karamanukyan, T. , & Edmands, S. (2014). Acclimation and adaptation to common marine pollutants in the copepod *Tigriopus californicus* . Chemosphere, 112, 465–471. 10.1016/j.chemosphere.2014.05.023 25048941

[jane70012-bib-0061] Tsui, M. T. K. , & Wang, W. (2005). Multigenerational acclimation of *Daphnia magna* to mercury: Relationships between biokinetics and toxicity. Environmental Toxicology and Chemistry, 24(11), 2927–2933. 10.1897/05-085R.1 16398130

[jane70012-bib-0062] Ullah, F. , Gul, H. , Tariq, K. , Desneux, N. , Gao, X. , & Song, D. (2020). Thiamethoxam induces transgenerational hormesis effects and alteration of genes expression in *Aphis gossypii* . Pesticide Biochemistry and Physiology, 165, 104557. 10.1016/j.pestbp.2020.104557 32359559

[jane70012-bib-0063] Vigneron, A. , Geffard, O. , Coquery, M. , François, A. , Quéau, H. , & Chaumot, A. (2015). Evolution of cadmium tolerance and associated costs in a Gammarus fossarum population inhabiting a low‐level contaminated stream. Ecotoxicology, 24(6), 1239–1249. 10.1007/s10646-015-1491-z 26003835

[jane70012-bib-0064] Völker, C. , Boedicker, C. , Daubenthaler, J. , Oetken, M. , & Oehlmann, J. (2013). Comparative toxicity assessment of Nanosilver on three daphnia species in acute, chronic and multi‐generation experiments. PLoS One, 8(10), e75026. 10.1371/journal.pone.0075026 24116021 PMC3792065

[jane70012-bib-0065] Walczyńska, A. , Franch‐Gras, L. , & Serra, M. (2017). Empirical evidence for fast temperature‐dependent body size evolution in rotifers. Hydrobiologia, 796(1), 191–200. 10.1007/s10750-017-3206-3

[jane70012-bib-0066] Weston, D. P. , Poynton, H. C. , Wellborn, G. A. , Lydy, M. J. , Blalock, B. J. , Sepulveda, M. S. , & Colbourne, J. K. (2013). Multiple origins of pyrethroid insecticide resistance across the species complex of a nontarget aquatic crustacean, *Hyalella azteca* . Proceedings of the National Academy of Sciences of the United States of America, 110(41), 16532–16537. 10.1073/pnas.1302023110 24065824 PMC3799301

[jane70012-bib-0067] Wistrand‐Yuen, E. , Knopp, M. , Hjort, K. , Koskiniemi, S. , Berg, O. G. , & Andersson, D. I. (2018). Evolution of high‐level resistance during low‐level antibiotic exposure. Nature Communications, 9(1), 1599. 10.1038/s41467-018-04059-1 PMC5913237 29686259

[jane70012-bib-0068] Xi, Y. , Huang, K.‐Q. , Pan, L. , Zhu, H. , Ge, Y. , Wen, X. , & Xiang, X. (2019). Rapid adaptation of *Brachionus angularis* (Rotifera) to invasion by *Brachionus calyciflorus* . Hydrobiologia, 844(1), 31–42. 10.1007/s10750-019-3959-y

[jane70012-bib-0069] Yue, W. , Mo, L. , & Zhang, J. (2021). Reproductive toxicities of 1‐ethyl‐3‐methylimidazolium bromide on *Caenorhabditis elegans* with oscillation between inhibition and stimulation over generations. Science of the Total Environment, 765, 144334. 10.1016/j.scitotenv.2020.144334 33385812

[jane70012-bib-0070] Zhang, W. , & Declerck, S. A. J. (2022). Reduced fertilization constitutes an important prezygotic reproductive barrier between two sibling species of the hybridizing *Brachionus calyciflorus* species complex. Hydrobiologia, 849(7), 1701–1711. 10.1007/s10750-022-04814-y

[jane70012-bib-0071] Zhu, H. , Huang, Z. Y. , Jiang, S. , Pan, L. , & Xi, Y. L. (2022). Rapid adaptation of *Brachionus dorcas* (Rotifera) to tetracycline antibiotic stress. Aquatic Toxicology, 245, 106126. 10.1016/j.aquatox.2022.106126 35228124

