# Supplementary material for: Adapting to an increasingly stressful environment: Experimental evidence for ‘micro‐evolutionary priming’
Source: J Anim Ecol. 2025 Feb 19;94(5):863–73. doi: 10.1111/1365-2656.70012 (PMC12056352; doi:10.1111/1365-2656.70012)
Supplement: Supplementary file 1 — Figure S1. The selection experiment was started with creating 6 populations consisting of an identical set of 50 clones. Figure S2. Percentage of females with sexual eggs in Control and Cu selected populations in response to the copper addition treatments of the common garden experiment. Figure S3. Copper concentration in the medium for rotifer populations in response to copper addition treatments. Table S1. Model selection based on AICc values. Table S2. General linear mixed effects models for population growth rate and fecundity. Table S3. Generalized linear mixed effects models (binomial distribution with log‐link function) for mortality (counts of dead versus alive rotifers) and sexual investment (counts of females with sexual eggs versus parthenogenetic eggs). Supplementary Methods 1. Methodological description of the Copper Concentration Assessment. [file JANE-94-863-s001.docx]

**Supplementary Information: Adapting to an increasingly stressful environment: experimental evidence for ‘micro-evolutionary priming’**

Shuwen Han^1^, Paul J. Van den Brink^2^ and Steven A.J. Declerck^1,3^

^1^ Department of Aquatic Ecology, Netherlands Institute of Ecology (NIOO-KNAW), P.O. Box 50, 6700AB Wageningen, the Netherlands

^2^ Department of Aquatic Ecology and Water Quality Management, Wageningen University, P.O. Box 47, 6700 AA Wageningen, The Netherlands

^3^ Department of Biology, Laboratory of Aquatic Ecology, Evolution and Conservation, KULeuven, Ch. de Beriotstraat 32, 3000 Leuven, Belgium


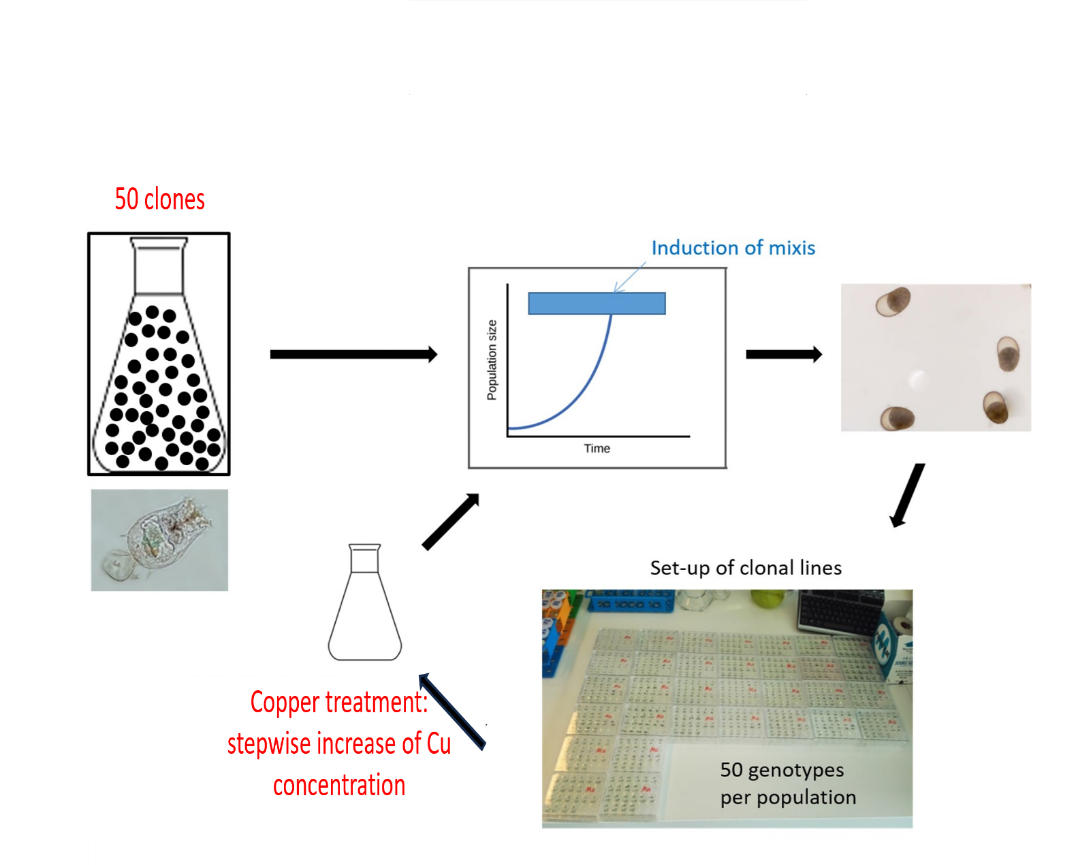


**Figure S1.** The selection experiment was started with creating 6 populations consisting of an identical set of 50 clones. Each of the populations went through six subsequent cycles of clonal growth followed by sexual reproduction. At the end of each cycle, sexually produced dormant propagules were hatched to obtain 50 clonal lines per population whereas the remainder were stored for later use. Each of the original populations was re-initiated by combining the clonal lines and a new cycle was started. At the beginning of each cycle, Cu was increased in the medium of the three populations belonging to the Cu addition treatment (from 30, 45, 55, 57.5, 60 to 62.5 µg Cu/L in Cycles 1 to 6, respectively). Modified from (Zhao *et al.* 2023).


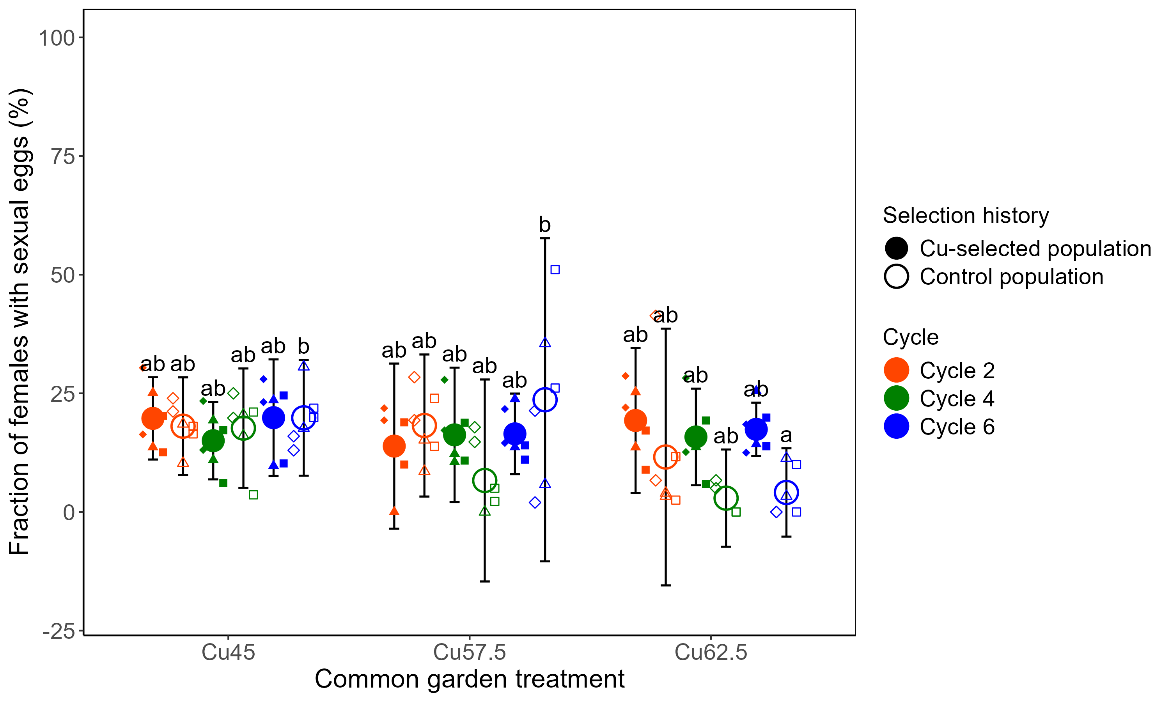


**Figure S2.** Percentage of females with sexual eggs in Control and Cu selected populations in response to the copper addition treatments of the common garden experiment. Selection history: Cu exposure history in the selection experiment; Cycle: cycle after which clones were extracted from the populations in the selection experiment. Symbols and error bars represent means and 95% confidence intervals across population replicates of the selection experiment. Letters denote differences according to post hoc Tuckey pairwise comparisons (alpha = 0.05). Small symbols represent individual clones where different symbol types (squares, triangles and diamonds) identify pairs of clones that originated from the same population in the selection experiment (see also Figure 3).


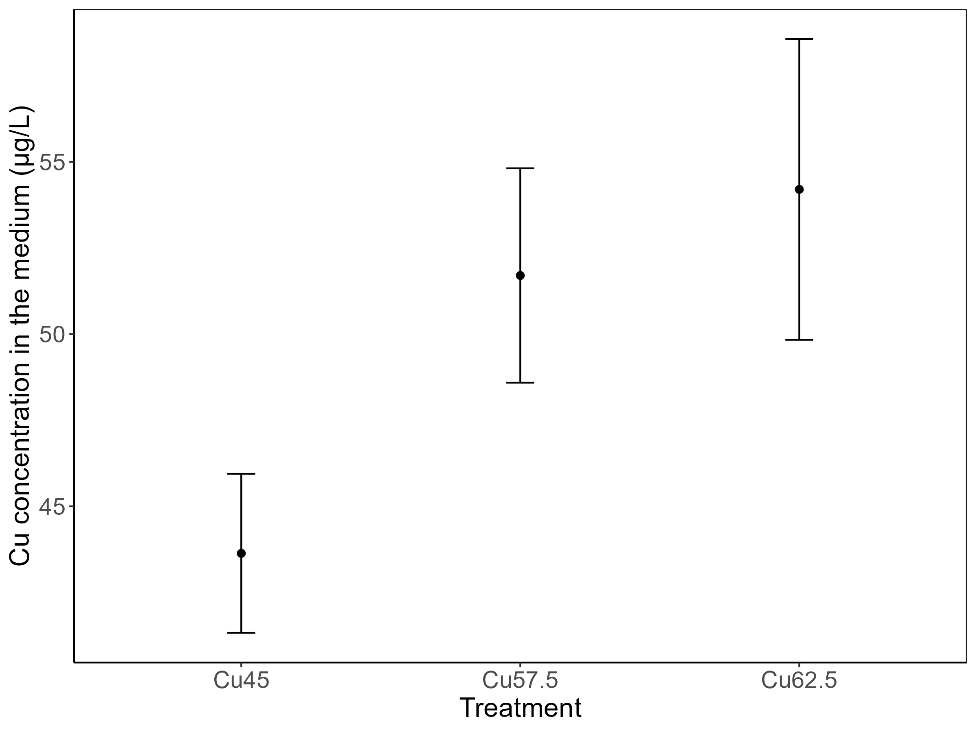


**Figure S3.** Copper concentration in the medium for rotifer populations in response to copper addition treatments. The medium samples were daily collected during a continuous period of five days. Error bars represent the 95% confidence interval.

**Table S1.** Model selection based on AICc values. Fixed effects were fitted by backward selection based upon AICc values calculated using ML estimation. Models with the lowest AICc (in bold) were selected for further analysis.

| **Dependent variable** | **Model type** | **Distribution** | **Clone†** |  | **Cu** | **SelHist** | **Cycle** | **Cu x SelHist** | **Cu x Cycle** | **SelHistx Cycle** | **Cu x SelHist x Cycle** | **K** | **AICc** | **ΔAICc** | **ML** | **AICcWt** | **LL** |  | **R^2^_marg_** | **R^2^_cond_** |
| --- | --- | --- | --- | --- | --- | --- | --- | --- | --- | --- | --- | --- | --- | --- | --- | --- | --- | --- | --- | --- |
| GR | mixed effects | normal | x |  | x | x | x | x | x | x | x | 21 | -209 | 0 | 1.000 | 1.000 | 131 |  | 0.958 | 0.96 |
|  |  |  |  |  | x | x | x | x |  |  |  | 11 | -167 | 42 | 0.000 | 0.000 | 96 |  |  |  |
|  |  |  |  |  | x | x |  | x |  |  |  | 9 | -159 | 50 | 0.000 | 0.000 | 90 |  |  |  |
|  |  |  |  |  | x | x |  |  |  |  |  | 7 | -40 | 169 | 0.000 | 0.000 | 27 |  |  |  |
|  |  |  |  |  | x | x | x |  |  |  |  | 9 | -39 | 169 | 0.000 | 0.000 | 30 |  |  |  |
|  |  |  |  |  |  |  |  |  |  |  |  |  |  |  |  |  |  |  |  |  |
| FEC | mixed effects | normal | x |  | x | x | x | x |  |  |  | 10 | -72 | 0 | 1.000 | 0.779 | 47 |  | 0.059 | 0.091 |
|  |  |  |  |  | x | x |  | x |  |  |  | 8 | -69 | 3 | 0.262 | 0.204 | 43 |  |  |  |
|  |  |  |  |  | x | x | x | x | x | x | x | 20 | -64 | 8 | 0.021 | 0.016 | 57 |  |  |  |
|  |  |  |  |  | x | x | x |  | x |  |  | 10 | -34 | 38 | 0.000 | 0.000 | 28 |  |  |  |
|  |  |  |  |  | x | x | x |  |  |  |  | 8 | -32 | 40 | 0.000 | 0.000 | 25 |  |  |  |
|  |  |  |  |  |  |  |  |  |  |  |  |  |  |  |  |  |  |  |  |  |
|  |  |  |  |  |  |  |  |  |  |  |  |  |  |  |  |  |  |  |  |  |
| Mortality | GLMM | Binomial  denominator | |  | x | x | x | x | x | x | x | 19 | 626 | 0 | 1.000 | 0.999 | -290 |  | 0.322 | 0.328 |
|  |  | (logit link) | x |  | x | x | x | x |  |  |  | 9 | 640 | 14 | 0.001 | 0.001 | -310 |  |  |  |
|  |  |  |  |  | x | x |  | x |  |  |  | 7 | 646 | 20 | 0.000 | 0.000 | -315 |  |  |  |
|  |  |  |  |  | x | x | x |  |  | x |  | 9 | 661 | 35 | 0.000 | 0.000 | -321 |  |  |  |
|  |  |  |  |  | x | x | x |  | x |  |  | 11 | 682 | 56 | 0.000 | 0.000 | -329 |  |  |  |
|  |  |  |  |  |  |  |  |  |  |  |  |  |  |  |  |  |  |  |  |  |
|  |  |  |  |  |  |  |  |  |  |  |  |  |  |  |  |  |  |  |  |  |
| Sexual eggs | GLMM | Binomial denominator | x |  | x | x |  | x |  |  |  | 7 | 616 | 0 | 1.000 | 0.750 | -300 |  | 0.033 | 0.07 |
|  |  | (logit link) |  |  | x | x | x | x |  |  |  | 9 | 618 | 2 | 0.328 | 0.246 | -299 |  |  |  |
|  |  |  |  |  | x | x | x | x | x | x | x | 19 | 626 | 10 | 0.006 | 0.004 | -290 |  |  |  |
|  |  |  |  |  | x |  |  |  |  |  |  | 4 | 632 | 16 | 0.000 | 0.000 | -312 |  |  |  |
|  |  |  |  |  | x | x |  |  |  |  |  | 5 | 634 | 18 | 0.000 | 0.000 | -312 |  |  |  |

Note: †: Random factor; K: number of estimated parameters; ML: model likelihood; AICcWt: model Akaike weights; LL: log-likelihood

**Table S2.** General linear mixed effects models for population growth rate and fecundity. Common garden Cu addition, Selection history and Cycle were specified as fixed factors, whereas Clone as random factor.

|  |  |  |  |  |  |  |  |
| --- | --- | --- | --- | --- | --- | --- | --- |
| **Dependent variable** | **Factor** | **SS** | **MS** | **NumDF** | **DenDF** | **F** | **P** |
| Population growth rate | Cu-treatment (Cu) | 4.99 | 2.50 | 2 | 60 | 417 | <0.001 |
|  | Selection history (SelHist) | 6.21 | 6.21 | 1 | 30 | 1037 | <0.001 |
|  | Cycle | 0.13 | 0.07 | 2 | 30 | 11 | <0.001 |
|  | Cu x SelHist | 2.59 | 1.29 | 2 | 60 | 216 | <0.001 |
|  | Cu x Cycle | 0.17 | 0.04 | 4 | 60 | 7 | <0.001 |
|  | SelHist x Cycle | 0.13 | 0.06 | 2 | 30 | 11 | <0.001 |
|  | Cu x SelHist x Cycle | 0.19 | 0.05 | 4 | 60 | 8 | <0.001 |
|  |  |  |  |  |  |  |  |
| Fecundity | Cu | 3.03 | 1.52 | 2 | 68 | 73 | <0.001 |
|  | SelHist | 0.89 | 0.89 | 1 | 32 | 43 | <0.001 |
|  | Cycle | 0.15 | 0.08 | 2 | 32 | 4 | 0.036 |
|  | Cu x SelHist | 1.24 | 0.62 | 2 | 68 | 30 | <0.001 |

**Table S3.** Generalized linear mixed effects models (binomial distribution with log-link function) for mortality (counts of dead versus alive rotifers) and sexual investment (counts of females with sexual eggs versus parthenogenetic eggs). Common garden Cu addition, Selection history and Cycle were specified as fixed factors, whereas Clone as random factor. Chisq: Type III Wald chisquare; df: degrees freedom. Bold P-values refer to significant effects (<0.05).

| **Dependent variable** | **Factor** | **Chisq** | **Df** | **P** |
| --- | --- | --- | --- | --- |
| Mortality | Intercept | 380 | 1 | 0.001 |
|  | Cu-treatment (Cu) | 76 | 2 | 0.001 |
|  | Selection history (SelHist) | 1 | 1 | 0.335 |
|  | Cycle | 15 | 2 | 0.001 |
|  | Cu x SelHist | 21 | 2 | 0.001 |
|  | Cu x Cycle | 4 | 4 | 0.378 |
|  | SelHist x Cycle | 4 | 2 | 0.167 |
|  | Cu x SelHist x Cycle | 8 | 4 | 0.076 |
|  |  |  |  |  |
| Frequency of females with sexual eggs | Intercept | 206 | 1 | 0.001 |
|  | Cu-treatment (Cu) | 3 | 2 | 0.237 |
|  | Selection history (SelHist) | 0 | 1 | 0.927 |
|  | Cu x SelHist | 20 | 2 | 0.001 |

**Supplementary methods 1: Methodological description of the Copper Concentration Assessment**

Due to the potential of copper to bind with both organic and inorganic substrates, rotifers may have experienced different copper concentrations than intended in the experimental treatments of the common garden experiment. The Copper Concentration Assessment aimed at measuring actual Cu concentrations experienced by rotifers under the same conditions as in the treatments of the common garden experiment.

The design of the experiment consisted of 9 experimental units, with three replicate rotifer populations being exposed to three copper addition treatments (45, 57.5 and 62.5 µg Cu/L). The execution of the experiment consisted of three phases: (1) the establishment of clonal lines, (2) an acclimation phase and (3) a collection phase.

(1) Establishment of clonal lines: From each of the three replicate populations with a 62.5 µg Cu/L selection history (SelHist_Cu62.5), we established three clonal lines from dormant propagules produced during the sixth cycle of the selection experiment.

(2) Acclimation phase: After an initial phase of population upscaling under food satiating conditions (*C. reinhardtii*; 1000 µmol C/L) in a copper free environment, we created nine experimental populations by transferring 2 randomly selected individuals of each of the nice clonal populations to wells with 9 ml of the food suspension. Subsequently, these cultures were further maintained by daily transferring 18 random individuals to a fresh food suspension, excluding males and females with resting eggs while gradually increasing copper levels until the final experimental target concentrations were reached (i.e. 45, 57.5 and 62.5 µg Cu/L). Subsequently, we monitored populations for a period of 5 days to ensure that daily population growth had stabilized.

(3) Collection phase: During this phase we continued the maintenance of the cultures in the same way as for the acclimation phase. After each daily transfer, the remaining culture medium was centrifuged for 30 minutes to remove the algae using a high-speed centrifuge (14,800 rpm) and the supernatant stored in a freezer at -20 °C For each experimental unit, this was daily repeated during a period of 5 days. At the end, all medium collected for a given unit was pooled, and filtered through a GF/F membrane filter (diameter: 47 mm; pore size; 0.45 µm) to remove any remaining algae and other particles. The filtered medium was stored at -20 °C until further analysis.

References:

Zhao, S. Y., Zhou, L., Chen, G., & Declerck, S. A. J. (2023). Rapidly evolving zooplankton in a salinizing world: To what extent does microevolutionary adaptation to one salt increase tolerance to another one? *Limnology and Oceanography*, *68(50), 2576-2586*. https://doi.org/10.1002/lno.12443
